# Deep learning-based susceptibility map-weighted imaging analysis of dorsal nigral hyperintensity as a surrogate for dopamine transporter positron emission tomography in patients with idiopathic rapid eye movement sleep behavior disorder

**DOI:** 10.3389/fneur.2026.1756028

**Published:** 2026-08-28

**Authors:** Beomseok Sohn, Jae Rim Kim, Hwan Heo, Soohwa Song, Eung Yeop Kim, Eun Yeon Joo

**Affiliations:** 1 Department of Radiology, Samsung Medical Center, Sungkyunkwan University School of Medicine, Seoul, Republic of Korea; 2 Department of Neurology, Chungnam National University Sejong Hospital, Sejong, Republic of Korea; 3 Heuron Co., Ltd., Seoul, Republic of Korea; 4 Department of Neurology, Samsung Medical Center, Sungkyunkwan University School of Medicine, Seoul, Republic of Korea

**Keywords:** artificial intelligence, deep learning, dopamine transporter PET, idiopathic REM sleep behavior disorder, susceptibility map-weighted imaging

## Abstract

Idiopathic REM sleep behavior disorder (iRBD) is a prodromal stage of alpha-synucleinopathies, and accurate identification of early dopaminergic dysfunction is clinically important. This study evaluated whether deep learning–based susceptibility map-weighted imaging (SMwI) can predict dopamine transporter (DAT) positron emission tomography (PET) abnormalities in patients with iRBD. We retrospectively included 74 patients who underwent both SMwI and ^18^F-FP-CIT PET and applied a deep learning model to detect the absence of dorsal nigral hyperintensity. Diagnostic performance was assessed at patient and hemisphere levels. Deep learning–based SMwI showed a sensitivity of 78%, specificity of 66.7%, and accuracy of 73% for predicting DAT PET abnormalities. Bilateral SMwI abnormalities strongly correlated with DAT PET positivity (24/26; 92.3%), whereas DAT PET abnormalities were present in 35.5% of patients with visually unremarkable SMwI. These findings indicate that AI-assisted SMwI provides moderate agreement with DAT PET and may serve as a practical triage or follow-up tool for identifying presynaptic dopaminergic dysfunction in iRBD, particularly where access to DAT PET is limited.

## Introduction

Clinical data alone are insufficient to accurately predict phenoconversion in patients with idiopathic rapid eye movement sleep behavior disorder (iRBD), a recognized prodromal stage of alpha-synucleinopathies, including Parkinson’s disease (PD) and dementia with Lewy bodies (1–3). Therefore, neuroimaging biomarkers have gained increasing attention because of their potential to detect early dopaminergic dysfunction in iRBD (4, 5). Among these imaging modalities, dopamine transporter (DAT) positron emission tomography (PET), including ^18^F-N-(3-fluoropropyl)-2β-carboxymethoxy-3β-(4-iodophenyl) nortropane PET (^18^F-FP-CIT PET), has specific advantages but is limited by high costs, radiation exposure, and availability only in specialized centers (6).

Magnetic resonance imaging (MRI)-based techniques have recently attracted interest as alternative noninvasive biomarkers for identifying early dopaminergic dysfunction (7, 8). Susceptibility map-weighted imaging (SMwI), an advanced iron-sensitive MRI technique, can be used to effectively visualize dorsal nigral hyperintensity (DNH) within the substantia nigra (SN) pars compacta (SNpc), a radiologic marker conventionally associated with the nigrosome-1 region (9). SMwI correlates closely with DAT PET findings, achieving high diagnostic accuracy for PD, with sensitivities and specificities exceeding 95% in several reports (10, 11). Given its proven utility in PD diagnosis, SMwI has potential as a diagnostic and prognostic tool for the prodromal stages of PD, specifically in patients with iRBD.

Despite its promising performance, visual assessment of DNH loss on SMwI remains challenging owing to inherent subjectivity, the necessity of extensive expertise, and substantial interobserver variability (12). Developments in deep learning (DL)-based artificial intelligence (AI) may offer a robust solution for overcoming these limitations by providing objective, reproducible, and reliable assessments of DNH integrity (13, 14). Studies have explored DL-based approaches for detecting nigral abnormalities on iron-sensitive MRI, including SMwI, demonstrating improved consistency and diagnostic performance compared with manual readings in PD cohorts (13, 15). Most prior DL-based SMwI studies have focused on differentiating patients with established Parkinson’s disease from healthy controls or other parkinsonian disorders. In contrast, patients with iRBD represent a prodromal population with subtler and more heterogeneous nigral imaging abnormalities, making automated detection substantially more challenging. Furthermore, relatively few studies have specifically evaluated whether DL-assisted SMwI can predict presynaptic dopaminergic dysfunction as assessed by DAT PET in iRBD.

Therefore, this study aimed to evaluate the diagnostic accuracy of DL-based AI-assisted SMwI in predicting presynaptic dopaminergic dysfunction as assessed by DAT PET in patients with iRBD.

## Materials and methods

### Patient selection

**Figure 1:**
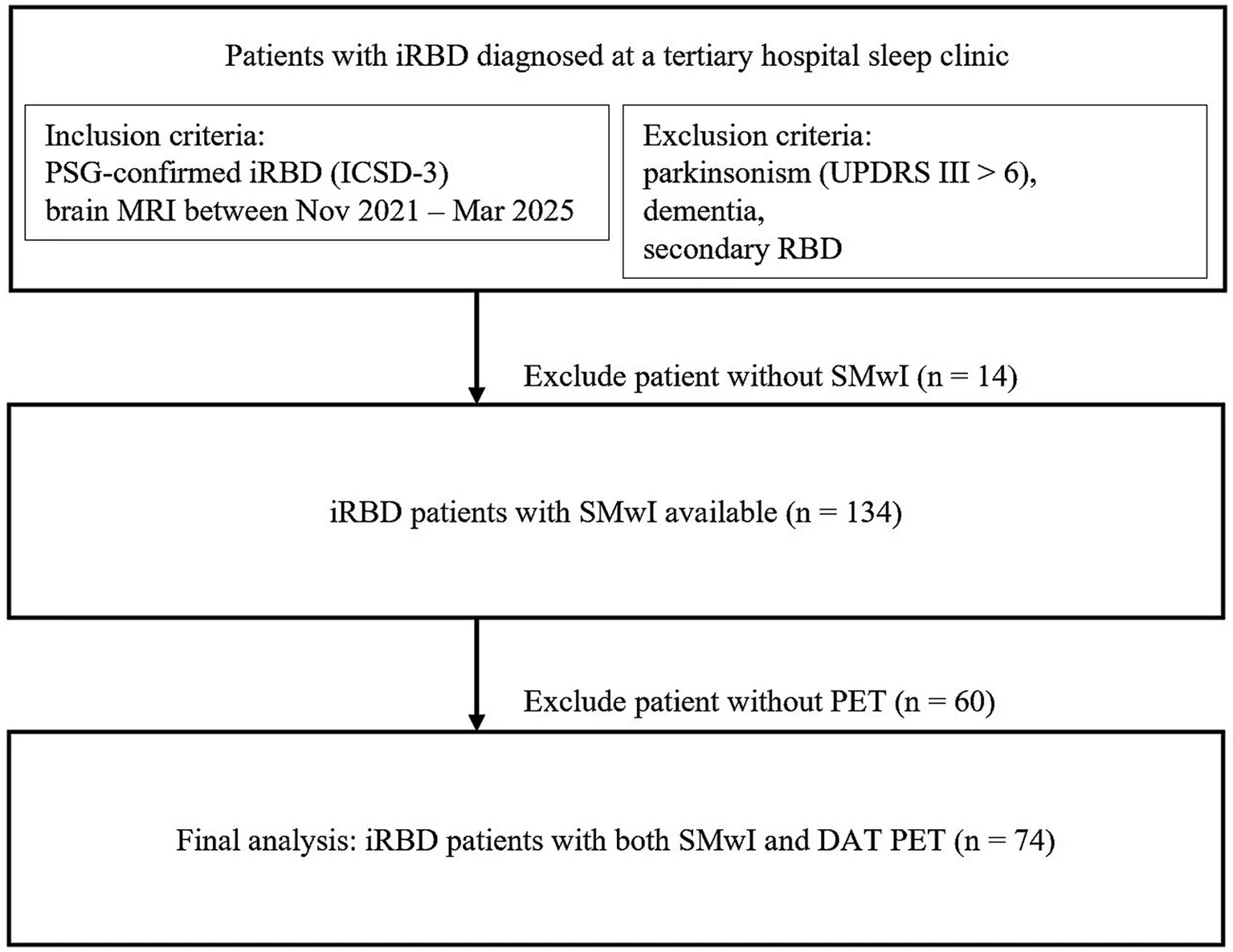
Patient enrollment flowchart. DAT, Dopamine transporter; PET, Positron emission tomography; SMwI, Susceptibility map-weighted imaging; MRI, Magnetic resonance imaging; iRBD, Idiopathic rapid eye movement sleep behavior disorder; PSG, Polysomnography; ICSD-3, International Classification of Sleep Disorders, third edition; UPDRS III, Unified Parkinson’s Disease Rating Scale part III.

This retrospective study was approved by the institutional review board (IRB No. 2023-02-043), and the requirement for informed consent was waived. We retrospectively reviewed patients diagnosed with iRBD at a tertiary hospital. The inclusion criteria were as follows: (1) overnight polysomnography; (2) clinical diagnosis of iRBD based on the International Classification of Sleep Disorders, third edition (16); and (3) brain MRI between November 2021 and March 2025. Patients were excluded if they exhibited clinical parkinsonism (defined as a Unified Parkinson’s Disease Rating Scale [UPDRS] Part III score >6), dementia, or secondary RBD (17). UPDRS Part III assessments were obtained during the initial neurological evaluation prior to neuroimaging acquisition. Among patients who met these criteria, we identified 148 who underwent brain MRI. Of these, 134 had available SMwI data and were included for further review. Of these, 60 did not undergo DAT PET imaging. Among the patients meeting the clinical and MRI inclusion criteria, only those who additionally underwent DAT PET imaging were included in the final MRI-PET comparison analyses (Figure 1).

### Image acquisition

SMwI data were acquired using a 3T MRI scanner (Ingenia CX, Philips Healthcare). The imaging parameters were as follows: repetition time, 48 ms; echo time, 14, 27, and 40 ms; echo spacing, 13 ms; flip angle, 20°; slice thickness, 1 mm; matrix size, 384 × 384; field of view, 192 × 192 mm. The imaging plane was an oblique coronal plane aligned parallel to a plane extending from the posterior commissure to the anterosuperior border of the pons and approximately perpendicular to the midbrain. Images were acquired as thin-section, non-contrast, gradient-echo sequences with three echoes. Quantitative susceptibility mapping (QSM) was computed using an iterative least-squares (iLSQR) method; QSM reconstructed using an iLSQR method was subsequently used for SMwI reconstruction to enhance visualization of DNH, according to previously established SMwI methodology (9, 18).

All patients underwent ^18^F-FP-CIT PET. Brain ^18^F-FP-CIT PET images were obtained using a PET/computed tomography (CT) scanner (Discovery STE, GE Healthcare, USA), 120 min after the injection of 185 MBq ^18^F-FP-CIT. PET images were acquired for 10 min in three-dimensional mode immediately after a brain CT scan for attenuation correction and image fusion. CT scanning was performed at 140 kVp and 50 mAs, with a slice thickness of 3.75 mm. The ^18^F-FP-CIT PET images were reconstructed using the VUE Point algorithm with a 128 × 128 matrix. Each ^18^F-FP-CIT PET scan was visually assessed and classified as either normal or abnormal by experienced nuclear medicine physicians, as part of routine clinical reporting.

### AI-based interpretation of SMwI

**Figure 2:**
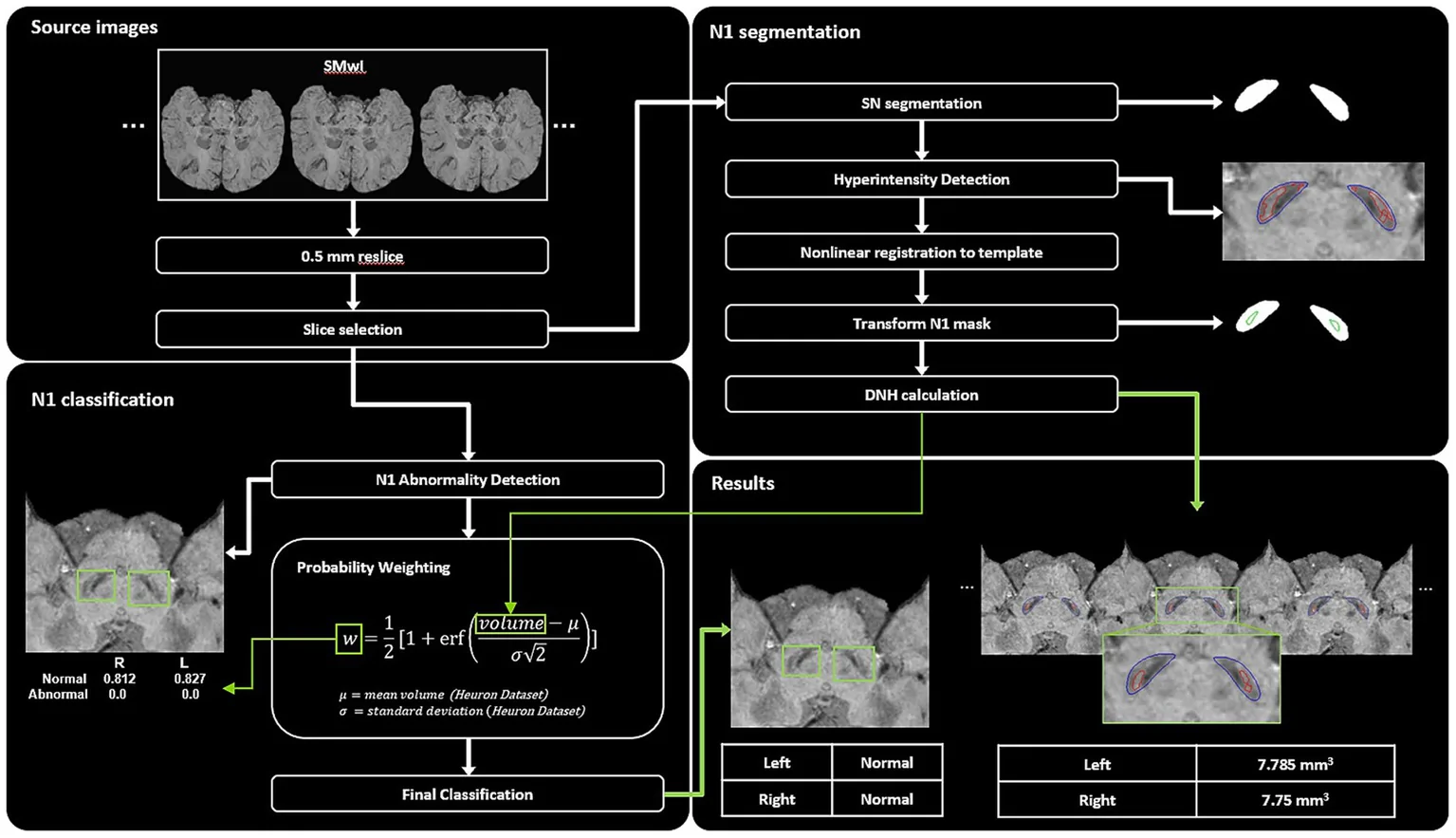
Automatic classification and segmentation of the dorsal nigral hyperintensity (DNH) using deep learning models. SMwI, susceptibility map-weighted imaging; SN, substantia nigra; DNH, dorsal nigral hyperintensity.

AI-generated interpretations were obtained using Heuron IPD software (Heuron Corporation, Seoul, Korea). The software integrates two independent deep learning models—segmentation of the SN and detection of DNH—to produce both binary classifications (normal vs. abnormal) and quantitative volume estimates. Five oblique coronal slices inferior to the red nucleus were automatically selected for analysis, and abnormality probabilities were weighted by corresponding DNH volumes to enhance classification accuracy (Figure 2). The algorithms have been previously described in detail by Suh et al. (13); additional implementation details, including model architectures, hyperparameters, and data augmentation strategies, are provided in the Supplementary methods. All SMwI datasets underwent visual quality assessment prior to AI analysis to confirm adequate visualization of the SN region and assess for significant imaging artifacts.

### Statistical analysis

Using DAT PET visual interpretation as the gold standard, we evaluated the performance of AI-based SMwI interpretations (binary predictions: normal or abnormal) by calculating sensitivity, specificity, positive predictive value (PPV), negative predictive value (NPV), and accuracy, along with the corresponding 95% confidence intervals (CIs). CIs were calculated using the Wilson score method without continuity correction. Performance was assessed at both the patient and hemisphere levels. For patient-level analysis, any PET abnormality (unilateral or bilateral) was considered positive. For side-based analyses, the left and right SN regions were evaluated independently, and SMwI abnormalities on each side were compared with the corresponding ipsilateral DAT PET findings. In cases with asymmetric or borderline DAT PET findings, the classification followed the dominant pattern of dopaminergic loss.

We assessed the predictive performance of the AI-derived quantitative DNH volume using receiver operating characteristic curve analysis. The optimal cutoff value was determined using Youden’s index; the corresponding diagnostic metrics (sensitivity, specificity, PPV, NPV, and accuracy) were computed at this threshold. All statistical analyses were performed using custom scripts in Python (version 3.9). Statistical significance was set at *p* < 0.05.

## Results

### Patient characteristics

Among the 134 patients with iRBD who underwent SMwI, 74 (52 men [70.3%], 22 women [29.7%]) underwent DAT PET. The mean age at MRI was 66.8 years (standard deviation [SD], 6.8 years). The interval between MRI and DAT PET ranged from −181 to 538 days (mean, 172.2 ± 167.2 days; median, 139.5 days; interquartile range, 46–292 days). Negative interval values indicate cases in which DAT PET was performed prior to MRI acquisition, whereas positive values indicate that MRI was performed before DAT PET. Detailed demographics, including age and sex, are presented in Table 1.

**Table 1:** Demographic and imaging characteristics of the study cohort.

Variable	Total (*n* = 74)	DAT PET positive (*n* = 41)	DAT PET negative (*n* = 33)	*p*-value
Demographics				
Age at MRI, mean ± SD (years)	66.8 ± 6.8	66.7 ± 6.1	67.1 ± 7.6	0.812
Sex, male/female, *n* (%)	52/22 (70.3/29.7)	26/15 (63.4/36.6)	26/7 (78.8/21.2)	0.237
MRI–PET interval, mean ± SD (days)	172.2 ± 167.2	142.1 ± 149.2	209.5 ± 182.6	0.092
AI-SMwI interpretation				
SMwI abnormal (AI-detected), *n* (%)	43 (58.1%)	32 (78.0%)	11 (33.3%)	<0.001
Bilateral abnormality, *n* (%)	26 (35.1%)	24 (58.5%)	2 (6.1%)	<0.001
Right DNH loss, *n* (%)	36 (48.6%)	30 (73.2%)	6 (18.2%)	<0.001
Left DNH loss, *n* (%)	33 (44.6%)	26 (63.4%)	7 (21.2%)	<0.001
Quantitative DNH volume				
Minimum of bilateral DNH volume, mean ± SD (mm^3^)	4.0 ± 2.3	3.1 ± 2.2	4.8 ± 2.1	0.009
Right DNH volume, mean ± SD (mm^3^)	4.85 ± 2.45	4.3 ± 2.3	5.6 ± 2.5	0.026
Left DNH volume, mean ± SD (mm^3^)	5.15 ± 2.50	4.5 ± 2.7	5.9 ± 2.1	0.015

DAT, dopamine transporter; PET, positron emission tomography; SMwI, susceptibility map-weighted imaging; MRI, magnetic resonance imaging; SD, standard deviation; AI, artificial intelligence; DNH, dorsal nigral hyperintensity.

### AI-based SMwI interpretation

AI analysis identified DNH abnormalities in 36 of 134 patients with iRBD (26.9%). Among the subgroup of 74 patients who underwent DAT PET, 43 (58.1%) demonstrated SMwI abnormalities. Of these, 26 exhibited bilateral involvement, whereas the remaining 31 were classified as having normal SMwI. In the hemisphere-level analysis, DNH abnormalities were detected in 36 right hemispheres (48.6%) and 33 left hemispheres (44.6%).

The quantitative SMwI-based DNH volumes measured using the AI model are summarized in Table 1. Mean right volume was 4.85 mm^3^ (SD, 2.45 mm^3^); mean left volume was 5.15 mm^3^ (SD, 2.50 mm^3^). The Shapiro–Wilk test confirmed a normal distribution of DNH volumes on both sides (histogram provided in Supplementary Figure 1).

### DAT PET imaging results

Of the 74 patients who underwent DAT PET, 41 (55.4%) were classified as abnormal and 33 (44.6%) as normal. Among the 41 DAT PET-positive patients, 15 showed symmetric bilateral abnormalities, nine had bilateral abnormalities with left predominance, two had right predominance, 12 had isolated left-sided abnormalities, and three had isolated right-sided abnormalities (Supplementary Table S1).

### Diagnostic performance of AI-based SMwI versus DAT PET

A patient-wise comparison of the AI-based SMwI predictions and DAT PET results is summarized in Table 2 as a 2 × 2 contingency table. Of the 41 DAT PET-positive patients, SMwI correctly identified 32 as abnormal (78%), whereas nine were missed (false negatives). Of the 33 DAT PET-negative patients, SMwI correctly identified 22 as normal but incorrectly indicated abnormalities in 11 cases (false positives).

**Table 2:** Diagnostic performance of artificial intelligence-based SMwI in predicting dopamine transporter PET abnormalities (Patient-Wise).

AI-based SMwI	PET abnormal	PET normal	Total
SMwI abnormal	32	11	43
SMwI normal	9	22	31
Total	41	33	74

PET, positron emission tomography; SMwI, susceptibility map-weighted imaging.

This resulted in a patient-wise diagnostic sensitivity, specificity, PPV, NPV, and accuracy of 78% (95% CI, 63.3–88.0), 66.7% (95% CI, 49.6–80.2), 74.4% (95% CI, 59.8–85.1), 71.0% (95% CI, 53.4–83.9), and 73.0% (95% CI, 61.9–81.8), respectively. The F1 score was 0.762.

**Figure 3:**
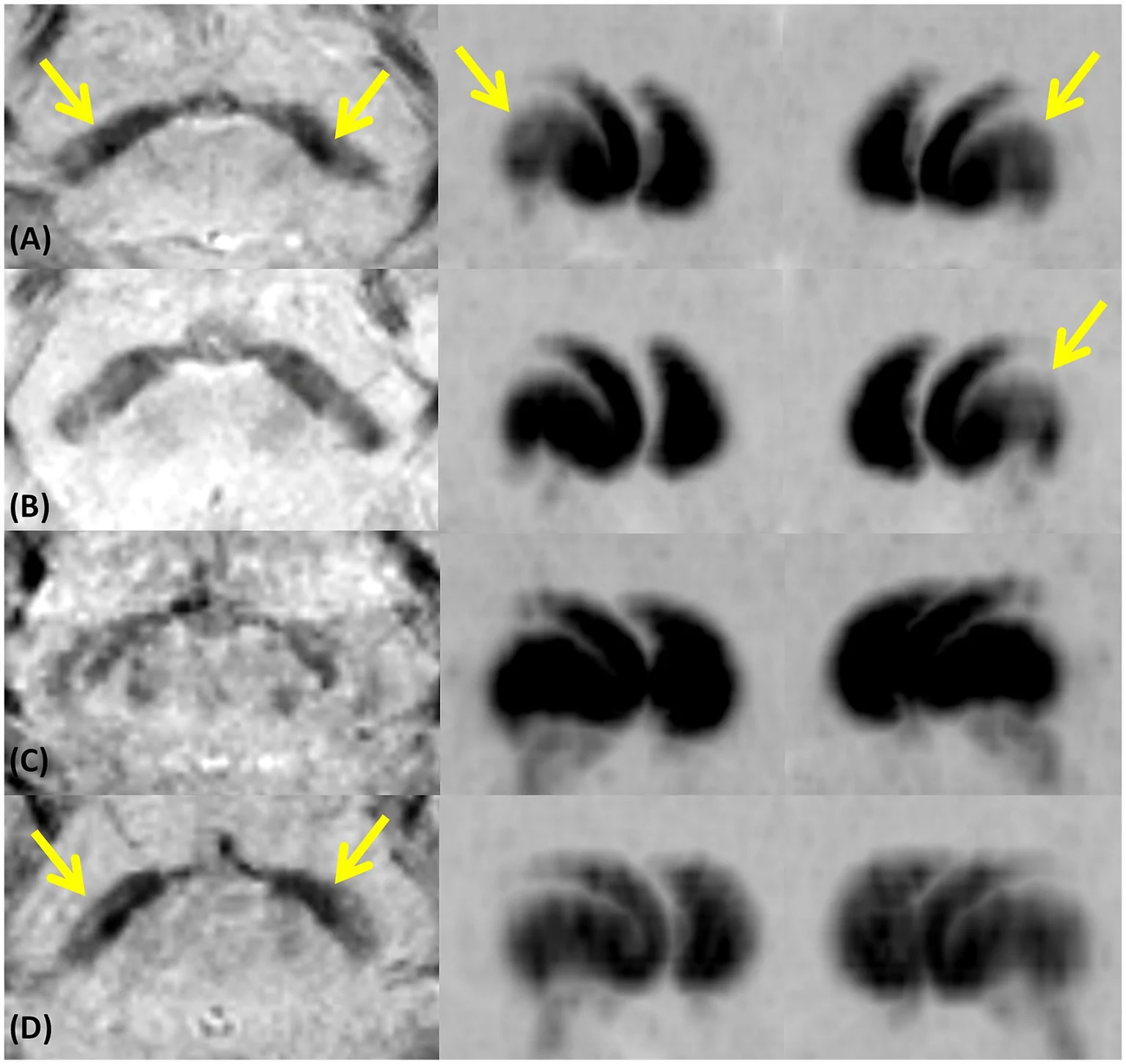
Representative concordant and discordant susceptibility map-weighted imaging (SMwI) and dopamine transporter (DAT) positron emission tomography images. **(A)** A 64-year-old woman with bilateral dorsal nigral hyperintensity (DNH) loss on SMwI (arrows) shows reduced DAT uptake bilaterally (true positive). **(B)** A 61-year-old woman with preserved DNH on SMwI exhibits a unilateral left DAT deficit (false negative). **(C)** A 62-year-old man with preserved DNH on SMwI demonstrates normal DAT uptake bilaterally (true negative). **(D)** A 72-year-old man with bilateral DNH loss on SMwI (arrow) shows preserved DAT uptake bilaterally (false positive).

Among the 11 false-positive SMwI cases, the image quality was rated as excellent in 10 cases; all underwent DAT PET after MRI. Nine false-negative cases also had excellent SMwI quality; eight underwent DAT PET within 200 days of MRI and one before MRI. Notably, eight of the nine false-negative cases exhibited unilateral DAT PET abnormalities, suggesting reduced SMwI sensitivity for detecting lateralized presynaptic deficits. Representative SMwI and DAT PET images of patients with bilateral abnormalities and unilateral mismatches are shown in Figure 3.

When DAT PET results were subclassified based on laterality and severity (symmetric bilateral, bilateral left predominant, bilateral right predominant, left only, and right only), SMwI demonstrated high concordance in cases with bilateral DAT abnormalities: 93.3% for symmetric bilateral and 100% for both bilateral left predominant and bilateral right predominant cases. Conversely, concordance was low in patients with unilateral DAT PET abnormalities, with match rates of 50.0% and 33.3% for left-only and right-only abnormalities, respectively (Supplementary Table S2).

### Side-based diagnostic performance

Side-based analysis comparing AI-based SMwI and DAT PET results showed a sensitivity, specificity, PPV, NPV, and accuracy of 73.2% (95% CI, 58.1–84.3), 81.8% (95% CI, 65.6–91.4), 83.3% (95% CI, 68.1–92.1), 71.1% (95% CI, 55.2–83.0), and 77.0% (95% CI, 66.3–85.1), respectively, in the right SN.

For the left SN, the corresponding values were sensitivity, 63.4% (95% CI, 48.1–76.4); specificity, 78.8% (95% CI, 62.2–89.3); PPV, 78.8% (95% CI, 62.2–89.3); NPV, 63.4% (95% CI, 48.1–76.4); and accuracy, 70.3% (95% CI, 59.1–79.5).

### Quantitative analysis of SMwI DNH volume

**Figure 4:**
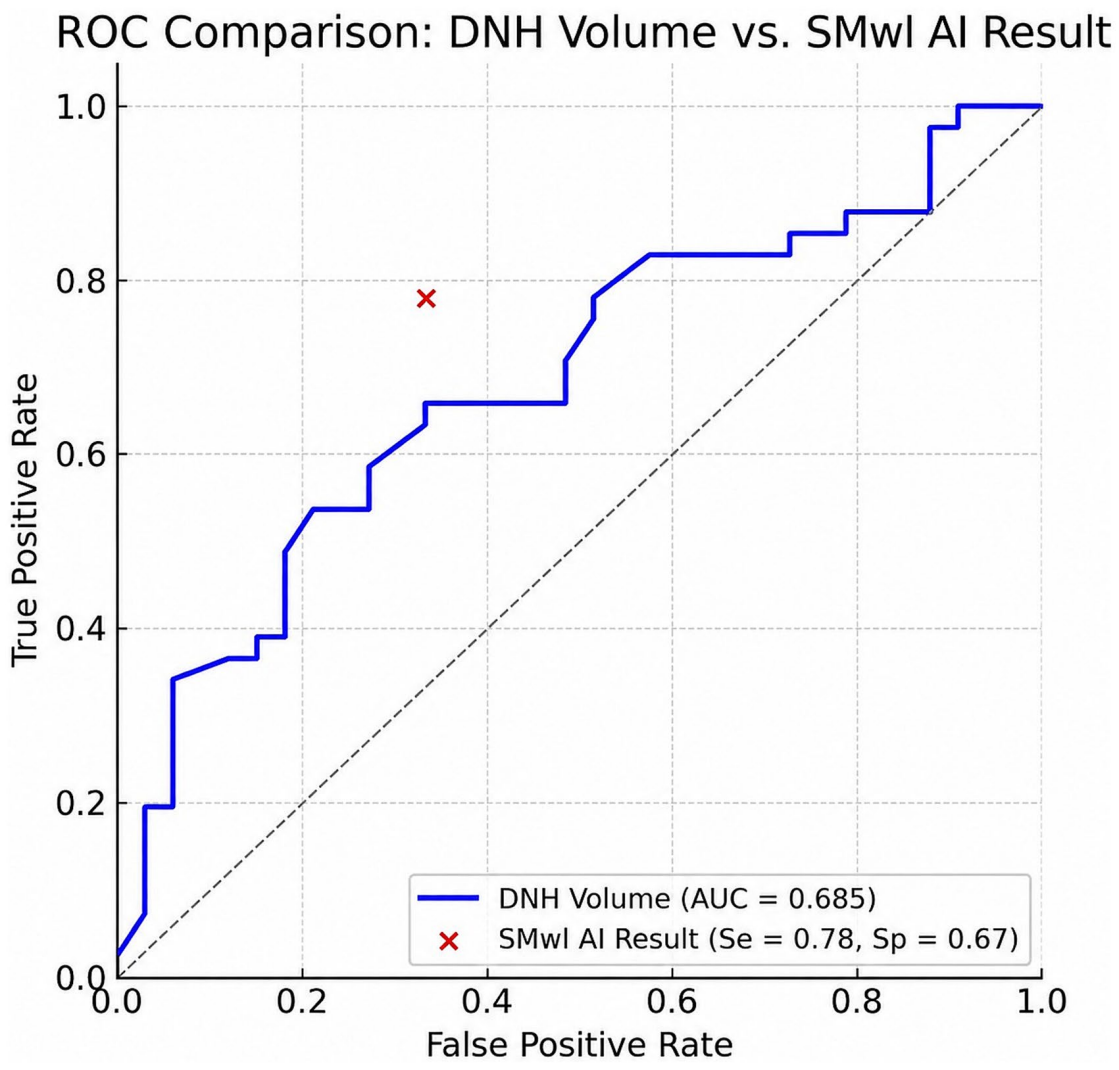
ROC comparison: DNH volume vs. SMwI AI result. Receiver operating characteristic (ROC) curve comparing the diagnostic performance of artificial intelligence (AI)-derived minimum dorsal nigral hyperintensity volume (blue curve) and binary susceptibility map-weighted imaging (SMwI) AI interpretation (red point) for predicting DAT PET abnormalities in patients with iRBD. The DNH volume yielded an AUC of 0.685. The red point represents the performance of the SMwI AI output (sensitivity, 78.0%; specificity, 66.7%).

Quantitative receiver operating characteristic analysis using the minimum DNH volume (i.e., the smaller volume between the right and left sides) against DAT PET abnormalities resulted in an AUC of 0.685 (Figure 4). Using a cutoff value of 3.88 mm^3^ determined by Youden’s index, the sensitivity, specificity, PPV, NPV, and accuracy was 65.9% (95% CI, 50.5–78.4), 66.7% (95% CI, 49.6–80.2), 71.1% (95% CI, 55.2–83.0), 61.1% (95% CI, 44.9–75.2), and 66.2% (95% CI, 54.9–76.0), respectively. The F1 score for this threshold was 0.684.

## Discussion

Here, we demonstrated that AI-assisted SMwI moderately predicted DAT PET abnormalities in patients with iRBD, with a sensitivity, specificity, and accuracy of 78%, 66.7%, and 73%, respectively. These findings support its potential use as a noninvasive triage tool for early dopaminergic dysfunction, although it is not intended to replace DAT PET. The diagnostic performance observed in this study suggests that fully automated AI-assisted SMwI analysis is feasible for identifying DAT PET abnormalities in patients with iRBD. Despite this promise, the moderate specificity (66.7%) indicated a considerable false-positive rate: 11 of 33 DAT PET-negative patients were classified as abnormal on SMwI. In these cases, the DNH volumes were consistently reduced, suggesting that SMwI might detect subtle structural alterations that are not necessarily accompanied by measurable dopaminergic dysfunction on DAT PET. Another possible explanation is the variable interval between MRI and DAT PET, which ranged from −181 to 538 days in this retrospective cohort. Therefore, temporal changes in dopaminergic status between imaging examinations may have contributed to discordant findings in some cases. Additionally, studies have reported incidental loss of the SNpc in neurodegenerative conditions, including Alzheimer’s disease, which may also account for false-positive DNH findings on SMwI (19, 20). These cases may represent nonspecific nigral structural alterations rather than definite prodromal synucleinopathy. Longitudinal follow-ups are warranted to determine whether SMwI abnormalities in DAT PET-negative patients can predict later dopaminergic decline or phenoconversion.

The majority of false-negative cases (eight of nine) exhibited unilateral abnormalities on DAT PET, with only one case involving bilateral changes. This suggests that SMwI tends to miss abnormalities in relatively early-stage cases where dopaminergic degeneration may be mild or localized. Studies on PD have shown that a loss of more than 50% of dopaminergic input is typically required before SMwI can detect DNH abnormalities (21). Given that iRBD represents a prodromal stage of PD, it is plausible that, in many false-negative cases, the extent of degeneration has not yet reached the threshold necessary for structural detection by SMwI. Another possible explanation for these false-negative findings is the “dying-back” phenomenon characteristic of PD, wherein dopaminergic degeneration begins at the presynaptic terminals in the striatum and progresses retrogradely toward the SNpc (22). This pathophysiological mechanism may account for cases in which DAT uptake is already reduced on DAT PET even when structural changes in DNH are not visible on SMwI. Third, DNH may serve as a more specific marker of phenoconversion than DAT PET. Although dopaminergic alterations may already begin in patients with iRBD, clinical phenoconversion may not occur until these changes reach the SNpc. Longitudinal follow-up studies are needed to validate these hypotheses.

An additional consideration when interpreting the observed MRI–PET discordance is that susceptibility-sensitive MRI and DAT PET may reflect different aspects of dopaminergic biology. While DAT PET primarily measures presynaptic dopamine transporter availability, susceptibility-sensitive MRI may capture broader pathological changes in the dopaminergic system. Previous studies have demonstrated associations between iron-sensitive MRI measures and PET markers of dopamine storage as well as dopamine D1 and D2/D3 receptor availability, suggesting that susceptibility-sensitive MRI may reflect aspects of dopaminergic pathology beyond dopamine transporter availability alone (23–25). Therefore, discordance between SMwI and DAT PET should not necessarily be interpreted as an error of either modality, but may instead reflect differences in the biological processes represented by the two imaging modalities, underscoring their complementary clinical utility.

These findings indicate that SMwI is unlikely to replace DAT PET for detecting ultra-early presynaptic dopaminergic dysfunction. Therefore, the principal value of SMwI may lie not in replacing DAT PET, but in serving as an accessible adjunct imaging biomarker in clinical practice. As an MRI-based and radiation-free imaging technique, SMwI is substantially more accessible than DAT PET in many clinical settings and may be more practical for longitudinal follow-up studies because repeated MRI acquisition is generally more feasible than repeated nuclear imaging. In addition, bilateral SMwI abnormalities demonstrated high concordance with DAT PET positivity in our cohort, suggesting potential utility as a marker of more advanced nigral involvement within the prodromal stage.

Our quantitative analyses using DNH volume yielded a lower diagnostic performance than that of the binary AI classification, with an AUC of 0.685. Even after applying optimized cutoffs using Youden’s index, neither patient-wise nor side-based volumetric analyses outperformed the AI binary model. This suggests that the DL algorithm leverages image features beyond absolute volume, potentially incorporating contrast gradients, regional intensity patterns, or edge sharpness that are not captured by simple volume metrics. This highlights the fundamental limitations of purely volumetric or quantitative approaches in the context of SMwI. As an iron-sensitive imaging technique, SMwI may reflect regional susceptibility variations that do not directly translate into size changes but instead manifest as qualitative signal loss or distortion. The clinical interpretation of the presence of DNH, whether by human readers or AI, often hinges more on the presence or absence of focal hyperintensity than on its extent, suggesting that the structural integrity of the DNH may be better assessed through qualitative features.

Future studies should explore the interpretability of the AI model by identifying the image characteristics that most strongly influence its predictions. Clarifying these internal model decision rules could help refine the diagnostic utility of SMwI, moving it beyond a black-box classifier toward a tool that reflects pathophysiological patterns in a clinically meaningful manner. Furthermore, integrating quantitative susceptibility measurements with recent pathological insights into nigrosome anatomy may elucidate the biological basis of susceptibility-sensitive MRI findings in prodromal synucleinopathies (26). This study had several limitations. First, its retrospective design limited the standardization of imaging intervals and clinical assessments. Although patients were longitudinally followed clinically, neurological examinations and imaging acquisition were not perfectly synchronized because of the retrospective study design. Another limitation of this study is the relatively broad interval between MRI and DAT PET acquisition. Progressive nigrostriatal degeneration may occur during the interval between imaging examinations, potentially influencing MRI-PET concordance in some patients. In particular, patients with subtle or evolving dopaminergic dysfunction may demonstrate abnormal DAT PET findings before structural abnormalities become sufficiently pronounced to be detected on SMwI. Conversely, some discordant cases may reflect interval progression occurring between the two imaging studies rather than true biological disagreement between modalities. Because of the retrospective study design, imaging acquisition timing was not standardized across patients. Accordingly, the observed diagnostic performance and ROC analyses should be interpreted with caution, as they reflect comparisons between examinations acquired at different stages of disease progression rather than simultaneous assessments of the same biological state. Prospective studies with standardized and near-contemporaneous MRI and DAT PET acquisition are warranted to determine the intrinsic diagnostic performance of AI-assisted SMwI. Third, DNH volumes were not normalized to intracranial volume (ICV), potentially introducing individual anatomical variability. However, the diagnostic process for DNH integrity—both by human raters and the AI model—relies primarily on the presence or absence of DNH rather than on absolute or normalized volume. Unlike global brain atrophy markers, DNH assessment is inherently qualitative; its clinical interpretation is not typically adjusted for ICV. Therefore, the lack of ICV normalization may have a limited impact on diagnostic performance in this specific context. In addition, only a small number of patients underwent overt phenoconversion during the available follow-up period, limiting further longitudinal subgroup analyses.

## Conclusion

In conclusion, AI-assisted SMwI interpretation demonstrated moderate concordance with DAT PET in patients with iRBD. Although insufficient to replace DAT PET, it may serve as a practical adjunct tool, particularly in settings with limited access to DAT imaging. Future longitudinal studies are warranted to determine whether SMwI abnormalities predict phenoconversion in prodromal synucleinopathies.

## Data Availability

The raw data supporting the conclusions of this article will be made available by the authors, without undue reservation.
