# Supplementary material for: Deep learning-based susceptibility map-weighted imaging analysis of dorsal nigral hyperintensity as a surrogate for dopamine transporter positron emission tomography in patients with idiopathic rapid eye movement sleep behavior disorder
Source: Front Neurol. 2026 Aug 28;17:1756028. doi: 10.3389/fneur.2026.1756028 (PMC13561941; doi:10.3389/fneur.2026.1756028)
Supplement: Supplementary file 1 [file Supplementary_file_1.DOCX]

**Deep Learning-Based Susceptibility Map-Weighted Imaging Analysis of Nigrosome 1 as a Surrogate for Dopamine Transporter Positron Emission Tomography in Patients with Idiopathic Rapid Eye Movement Sleep Behavior Disorder**

**Supplementary Materials**

**Supplementary Methods: [SMWI Data Acqusition]**

To scan images of the nigrosome-1 region, situated beneath the inferior pole of the red nucleus, oblique coronal imaging was employed(Sung et al., 2016). To enhance the depiction of the nigrosome-1 area, we used a specialized image processing method known as SMwI(Gho et al., 2014; Nam et al., 2017). Imaging was conducted using a 3D Gradient Echo (GRE) sequence with three echoes. Subsequently, Quantitative Susceptibility Mapping (QSM), calculated through an iterative least-square (iLSQR) method, was used to create masks for reconstructing the SMwI images. The settings for the 3T scanner included a repetition time (TR) of 49 ms, a minimum echo time (TE) of 14 ms, a maximum TE of 40 ms, with three echoes, an echo spacing of 13 ms, a flip angle of 20°, a slice thickness of 1 mm, a matrix size of 384 × 384, a field of view (FOV) of 192 × 192.

**Supplementary Methods: [AI-based Interpretation of SMwI]**

AI-generated interpretations were obtained using Heuron IPD software (Heuron Corporation, Seoul, Korea ). The software employs two independent AI models to analyze the SMwI images integrating the output of each model to estimate nigrosome-1(N1) abnormalities. Using the SparseInst segmentation model and the SMwI template, the volume of the N1 was computed. (Dorsal nigral hyperintensity, DNH). YOLOX object detection model was used to automatically select and analyze five slices covering the substantia nigra (SN) on each side (left and right), estimating the probability of N1 abnormalities. For each slice, if the probability of abnormality exceeds a predefined threshold (0.2, determined by internal validation), the corresponding DNH volume is used as a weighting factor. 450 participants (210 with IPD and 240 individuals in the control group) from four hospitals were used for training the detection and segmentation models(Suh et al., 2024). The annotation data required for training each model were manually generated by an experienced neurologist.

The SMwI data were uniformly resliced at a resolution of 0.5 mm. From these resliced images, five slices located inferior to the red nucleus (RN) were selected, as the N1 region is situated below the inferior border of the RN and within the SN(Sung et al., 2021). The selected slices were then used as input for the AI models (Figure #). To segment the N1 region, the SN was first segmented. In the SN segmentation step, we used the SparseInst model (Cheng et al., 2022). The model was trained using a ResNet(He et al., 2016) backbone and optimized with the AdamW optimizer, configured with a learning rate of 5×10⁻⁵ and a batch size of 16. To effectively address class imbalance and improve boundary delineation, we incorporated a combination of loss functions: focal loss, dice loss, and binary cross-entropy (BCE). To enhance the generalizability of the model, the training dataset was augmented using various techniques such as multi-scale resizing random brightness, contrast, horizontal flip, and rotation. Within the SN region predicted by the segmentation model, pixel values were binarized to highlight hyperintense areas. This step was performed to facilitate accurate identification of the N1 region and these areas were defined as the N1 region of interest. Subsequently, we conducted SN mask-based nonlinear registration to precisely quantify the N1 region. For registration, the SN mask from the SMwI template(Park et al., 2023; Sung et al., 2021) was used as the fixed data. An experienced neurologist annotated the SN and N1 region in the SMwI template using previously validated SMwI methodology(Suh et al., 2024). The SN masks predicted by the segmentation model for each slice were used as the moving data. The overlapping areas between the transformed N1 mask and the N1 region of interest were defined as the N1 region. The volume of DNH was defined by summing the calculated N1 region across all slices.

To evaluate N1 abnormalities within the substantia nigra, a deep learning–based detection pipeline was developed. First, we used the YOLOX model(Ge et al., 2021) to classify N1 abnormalities. The model utilized a CSP-Darknet backbone, an SGD optimizer (learning rate 0.01, batch size 8), and was trained with BCE and IOU loss functions for classification and localization, respectively. Augmentations such as multi-scale input resizing, mosaic, mixup, color jitter, and horizontal flipping were applied to enhance training robustness. The object detection model outputs a probability value reflecting the likelihood of a specific object, based on learned features during training. To enhance classification accuracy, when the probability of abnormality from the detection model exceeded 0.2, the DNH volume was used as a weighting factor. The probabilities weighted by the DNH volume were defined as weighted probabilities and were subsequently used to classify abnormalities within the N1 region.

**Table S1.** **Distribution patterns of DAT PET abnormalities in patients with idiopathic rapid eye movement sleep behavior disorder.**

| **Pattern of DAT PET abnormality** | **n (%)** |
| --- | --- |
| Symmetric bilateral | 15 (36.6%) |
| Bilateral, left predominant | 9 (22.0%) |
| Bilateral, right predominant | 2 (4.9%) |
| Unilateral, left | 12 (29.3%) |
| Unilateral, right | 3 (7.3%) |
| Total | 41 (100%) |

DAT, dopamine transporter; PET, positron emission tomography.

**Table S2. Concordance between SMwI and DAT PET results stratified by laterality of dopaminergic abnormality.**

| **DAT PET Pattern** | **No. of Patients** | **SMwI-Concordant Cases** | **Match Rate (%)** |
| --- | --- | --- | --- |
| Symmetric bilateral | 15 | 14 | 93.3 |
| Bilateral-left predominant | 9 | 9 | 100.0 |
| Bilateral-right predominant | 2 | 2 | 100.0 |
| Unilateral left-only | 12 | 6 | 50.0 |
| Unilateral right-only | 3 | 1 | 33.3 |

DAT, dopamine transporter; PET, positron emission tomography; SMwI, susceptibility map-weighted imaging.

**Supplementary Figure 1.** Distribution of nigrosome 1 volumes in patients with idiopathic rapid eye movement sleep behavior disorder.

Histograms of right and left nigrosome 1 volumes measured by susceptibility map-weighted imaging. Volume values followed a normal distribution based on the Shapiro–Wilk test.

**References**

Cheng, T., Wang, X., Chen, S., Zhang, W., Zhang, Q., Huang, C., Zhang, Z., & Liu, W. (2022). Sparse Instance Activation for Real-Time Instance Segmentation. 4433–4442. https://openaccess.thecvf.com/content/CVPR2022/html/Cheng_Sparse_Instance_Activation_for_Real-Time_Instance_Segmentation_CVPR_2022_paper.html

Ge, Z., Liu, S., Wang, F., Li, Z., & Sun, J. (2021). YOLOX: Exceeding YOLO Series in 2021 (arXiv:2107.08430). arXiv. https://doi.org/10.48550/arXiv.2107.08430

Gho, S.-M., Liu, C., Li, W., Jang, U., Kim, E. Y., Hwang, D., & Kim, D.-H. (2014). Susceptibility map-weighted imaging (SMWI) for neuroimaging. Magnetic Resonance in Medicine, 72(2), 337–346. https://doi.org/10.1002/mrm.24920

He, K., Zhang, X., Ren, S., & Sun, J. (2016). Deep Residual Learning for Image Recognition. 770–778. https://openaccess.thecvf.com/content_cvpr_2016/html/He_Deep_Residual_Learning_CVPR_2016_paper.html

Nam, Y., Gho, S.-M., Kim, D.-H., Kim, E. Y., & Lee, J. (2017). Imaging of nigrosome 1 in substantia nigra at 3T using multiecho susceptibility map-weighted imaging (SMWI). Journal of Magnetic Resonance Imaging, 46(2), 528–536. https://doi.org/10.1002/jmri.25553

Park, S., Sung, Y. H., Kim, W. R., Noh, Y., & Kim, E. Y. (2023). Correlation Between Neuromelanin-Sensitive MRI and 18 F-FP-CIT PET in Early-Stage Parkinson’s Disease: Utility of a Voxel-Wise Analysis by Using High-Spatial-Resolution MRI. Journal of Clinical Neurology, 19(2), 156. https://doi.org/10.3988/jcn.2022.0147

Suh, P. S., Heo, H., Suh, C. H., Lee, M. O., Song, S., Shin, D. H., Jo, S. Y., Chung, S. J., Heo, H., Shim, W. H., Kim, H. S., Kim, S. J., & Kim, E. Y. (2024). Deep learning-based algorithm for automatic quantification of nigrosome-1 and Parkinsonism classification using susceptibility map-weighted MRI. American Journal of Neuroradiology. https://doi.org/10.3174/ajnr.A8585

Sung, Y. H., Noh, Y., & Kim, E. Y. (2021). Early-stage Parkinson’s disease: Abnormal nigrosome 1 and 2 revealed by a voxelwise analysis of neuromelanin-sensitive MRI. Human Brain Mapping, 42(9), 2823–2832. https://doi.org/10.1002/hbm.25406

Sung, Y. H., Noh, Y., Lee, J., & Kim, E. Y. (2016). Drug-induced Parkinsonism versus Idiopathic Parkinson Disease: Utility of Nigrosome 1 with 3-T Imaging. Radiology, 279(3), 849–858. https://doi.org/10.1148/radiol.2015151466
